# Targeted next generation sequencing as a tool for precision medicine

**DOI:** 10.1186/s12920-019-0527-2

**Published:** 2019-06-03

**Authors:** Markus Gulilat, Tyler Lamb, Wendy A. Teft, Jian Wang, Jacqueline S. Dron, John F. Robinson, Rommel G. Tirona, Robert A. Hegele, Richard B. Kim, Ute I. Schwarz

**Affiliations:** 1 Division of Clinical Pharmacology, Department of Medicine, Western University, London Health Sciences Centre - University Hospital, 339 Windermere Road, London, ON N6A 5A5 Canada; 2 Department of Physiology and Pharmacology, Western University, Medical Sciences Building, Room 216, London, ON N6A 5C1 Canada; 3 Robarts Research Institute, Western University, 1151 Richmond St. N, London, ON N6A 5B7 Canada

**Keywords:** Targeted exome sequencing, Next generation sequencing, Pharmacogenes, Copy number variation, In silico prediction

## Abstract

**Background:**

Targeted next-generation sequencing (NGS) enables rapid identification of common and rare genetic variation. The detection of variants contributing to therapeutic drug response or adverse effects is essential for implementation of individualized pharmacotherapy. Successful application of short-read based NGS to pharmacogenes with high sequence homology, nearby pseudogenes and complex structure has been previously shown despite anticipated technical challenges. However, little is known regarding the utility of such panels to detect copy number variation (CNV) in the highly polymorphic cytochrome P450 (*CYP) 2D6* gene, or to identify the promoter (TA)_7_ TAA repeat polymorphism UDP glucuronosyltransferase (UGT) 1A1*28. Here we developed and validated PGxSeq, a targeted exome panel for pharmacogenes pertinent to drug disposition and/or response.

**Methods:**

A panel of capture probes was generated to assess 422 kb of total coding region in 100 pharmacogenes. NGS was carried out in 235 subjects, and sequencing performance and accuracy of variant discovery validated in clinically relevant pharmacogenes. *CYP2D6* CNV was determined using the bioinformatics tool CNV caller (VarSeq). Identified SNVs were assessed in terms of population allele frequency and predicted functional effects through in silico algorithms.

**Results:**

Adequate performance of the PGxSeq panel was demonstrated with a depth-of-coverage (DOC) ≥ 20× for at least 94% of the target sequence. We showed accurate detection of 39 clinically relevant gene variants compared to standard genotyping techniques (99.9% concordance), including *CYP2D6* CNV and UGT1A1*28. Allele frequency of rare or novel variants and predicted function in 235 subjects mirrored findings from large genomic datasets. A large proportion of patients (78%, 183 out of 235) were identified as homozygous carriers of at least one variant necessitating altered pharmacotherapy.

**Conclusions:**

PGxSeq can serve as a comprehensive, rapid, and reliable approach for the detection of common and novel SNVs in pharmacogenes benefiting the emerging field of precision medicine.

**Electronic supplementary material:**

The online version of this article (10.1186/s12920-019-0527-2) contains supplementary material, which is available to authorized users.

## Background

Rapid identification of genetic variation contributing to therapeutic drug response or adverse effects is essential for implementation of individualized pharmacotherapy [1]. Many gene-drug associations are now recognized as clinically relevant, particularly those involving genes encoding drug metabolizing enzymes, membrane transporters, and certain drug targets, which together are often referred to as pharmacogenes [2]. Clinical guidelines have been developed for drugs with the strongest level of evidence of utility for pharmacogenetic testing in patients. For instance, the Clinical Pharmacogenetics Implementation Consortium (CPIC), an international expert group, documents the available evidence and provides recommendations for clinicians on genotype-based drug therapy [3]. CPIC guidelines have now been reported for more than 35 drugs including the anticoagulant warfarin [4, 5], the antiplatelet agent clopidogrel [6, 7], the cholesterol- lowering medication simvastatin [8], chemotherapeutics such as thiopurines (azathioprine and mercaptopurine) [9, 10], tamoxifen [11], and fluoropyrimidines [12], as well as the antiretroviral therapeutics abacavir [13] and atazanavir [14]. As well, many pharmacogenetic biomarkers have been incorporated in drug labels by the US Food and Drug Administration [15] and the European Medicines Agency [16].

Earlier research evaluated common functional variation in pharmacogenes, while more recent large-scale whole genome or exome sequencing studies revealed that humans harbor a large number of rare, potentially deleterious variants in many of the same genes [17–20]. Specifically, the analysis of sequencing data for 146 pharmacogenes combining about 7500 individuals of the Exome Sequencing Project (ESP) [21] and the 1000 Genomes Project (1000G) [22] indicated that more than 90% of all recorded single nucleotide variants (SNVs) were rare with a minor allele frequency (MAF) below 1%, and that 30–40% of the predicted functional variability was associated with these rare variants [17]. Recent studies also support that rare SNVs in drug processing or drug target genes significantly contribute to interpatient differences in drug disposition and response beyond established common genetic predictors as shown for cytochrome P450 (*CYP*) *2C9* and warfarin dose requirement [23, 24] and solute carrier organic anion transporter (*SLCO) 1B1* and methotrexate clearance and toxicity [25].

Next-generation sequencing (NGS) refers to rapid, high-throughput technologies that enable massively parallel DNA sequencing of entire human genomes, exomes or coding exons of select genes [26]. Targeted exome NGS capture panels are gaining popularity for pharmacogenetic testing due to their time- and cost-effectiveness, and ability to simultaneously detect common and rare genetic variation [27, 28]. Despite anticipated technical challenges for the application of short-read based NGS to genes with high sequence homology, nearby pseudogenes and complex structure [29–31], these limitations might be overcome through careful probe design (i.e. target enrichment [32]) combined with advanced bioinformatics approaches as suggested by previous reports [27, 28, 33]. However, little is currently known regarding the utility of such panels to detect copy number variation (CNV) in the highly polymorphic *CYP2D6* gene, or to identify the promoter (TA)_7_ TAA repeat polymorphism UDP glucuronosyl-transferase (UGT) 1A1*28, two common polymorphisms known to affect enzymatic activity and alter dose requirements for substrate drugs [34, 35]. Therefore, we created a NGS-based exome capture panel (PGxSeq) capable of detecting clinically established as well as novel genetic variation with potential implications in drug disposition and response. We applied our PGxSeq panel to 1) evaluate the sequencing performance achieved with the utilized target- enrichment strategy, 2) determine the accuracy of variant discovery in clinically relevant pharmacogenes compared to traditional genotyping methods including *CYP2D6* CNV and UGT1A1*28, and 3) evaluate the identified variation with respect to population allele frequencies and predicted functional effects.

## Methods

### Sample collection

**Fig. 1:**
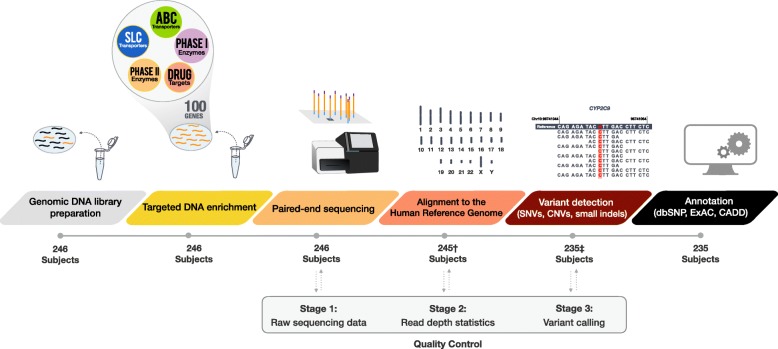
PGxSeq sample and data processing workflow (*n* = 246). Eleven subjects were excluded from variant analysis due to low read count (†; *n* = 1) and high GC content (‡; *n* = 10). All clip art depicted in this Figure has been created by the authors

Genomic DNA (gDNA) was obtained from venous blood samples of 246 Caucasian subjects (220 adult and 26 pediatric patients) following written informed consent. Studies were approved by the Research Ethics Board of Western University, London, Canada. A flow diagram of the sample and subsequent data processing can be found in Fig. 1.

### Gene selection, capture probe design and enrichment method

We used the Nextera Rapid Capture Custom Enrichment Kit (Illumina, San Diego, CA) for the enrichment of coding regions of 100 genes encoding major cytochrome P450 (CYP) enzymes, phase II conjugation enzymes, drug transporters of the solute carrier (SLC) and ATP binding cassette (ABC) families as well as regulatory proteins of relevance to variability in drug ADME (absorption, distribution, metabolism, excretion) and response including regions encompassing 14 known functional promoter or intronic SNVs such as UGT1A1*28, CYP3A5*3, and CYP2D6*41 (Additional file 1: Table S1 and S2).

A total of 10,207 capture probes (80 bp) were custom- designed using the Illumina Design Studio (Illumina, San Diego, CA) comprising 722 kilobases (kb) of sequence per sample (Genomic coordinates in Additional file 1: Table S1). Exons of all coding isoforms were targeted for selected genes including 300 bp intronic (flanking each exon) and 250 bp of 5′ and 3′ untranslated regions (UTR). Known functional non-coding variants were separately targeted if not captured otherwise (Additional file 1: Table S2). Chromosomal coordinates were obtained from University of California Santa Cruz (UCSC) genome browser using the GRCh37/hg19 human genome assembly. DNA library preparation and subsequent target-capture sequencing was conducted at the London Regional Genomics Center, London, Ontario, as previously described [36]. Briefly, DNA samples were processed in 13 runs in batches of 12 or 24 samples (referred to as sequencing cluster). After serial dilutions, DNA was adjusted to a final concentration of 5.0 ± 1 ng/μl using the Qubit DNA kit (Invitrogen, Eugene, OR). DNA was enzymatically fragmented, polymerase chain reaction (PCR)-amplified with individual sample barcodes, equimolar pooled, hybridized to the biotinylated capture probes, pooled using streptavidin beads, and PCR-amplified again to select the final target sequence. Resulting libraries were quantified, and loaded on to a standard flow-cell on the Illumina MiSeq Sequencer (Illumina, San Diego, CA) using 2 × 300 bp or 2 × 150 bp paired-end chemistry.

### Base calling, sequence alignment and variant detection

Prior to the alignment of reads to the reference genome, sequencing performance metrics were assessed (Fig. 1). Paired-end sequenced reads were separated according to sample-specific barcodes and sequencing data downloaded as FASTQ files that were further assessed with the quality control tool, FastQC [37], including read count, base quality across reads (also Phred score, Q; describes the probability of a sequencing error as a measure of base call accuracy), and guanine and cytosine (GC) content per sequence [38].

Alignment of sequencing reads and variant calling were performed using the CLC Bio Genomics Workbench 9.0 (CLC Bio, Aarhus, Denmark) through a custom automated workflow. FASTQ files were imported and mapped to the reference human genome (GRCh37/hg19 build). Using default algorithms (i.e. Local Realignment and Remove Duplicate Mapped Reads), initial read mapping was further optimized around insertion-deletion mutations (indels) and PCR duplicates removed.

Depth-of-coverage (DOC; also coverage) was defined as the number of reads mapped to a genomic position following alignment of sequenced reads and removal of duplicate reads. Reads that were non-specific matches (mapped to more than one location of hg19 reference genome) or missing the paired read were excluded from this calculation. For every subject, a Coverage Summary Report along with a base-by-base Coverage Table were exported. Coverage analysis was restricted to coding regions including 10 bp before and after each exon, and 250 bp of 3′ and 5’UTR. To detect samples with substantial regions of low coverage, we expressed coverage as percentage of the 422 kb target sequence with a DOC ≥1×, ≥ 10×, ≥ 20×, and ≥ 30× (Table 1). Subjects with more than 20% of their target sequence below 10× were excluded from variant analysis. Coverage was also assessed at the gene level and by sequencing cluster (*n* = 12 or 24).

**Table 1:** PGxSeq performance by sequencing cluster

	12-plex	24-plex
Subjects, n	52	183
Average reads (duplicates removed), per sample	1.01 M	0.54 M
DOC, mean (median)	213-fold ª (207-fold) ª	87.2-fold ª (84-fold) ª
Bases with mean DOC ≥1×, %	98.7 ^b^	98.6 ^b^
Bases with mean DOC ≥10×, %	98.0 ^b^	96.8 ^b^
Bases with mean DOC ≥20×, %	97.4 ^b^	94.3 ^b^
Bases with mean DOC ≥30×, %	96.8 ^b^	90.9 ^b^

*DOC* depth of coverage

ª Calculated across the 422 kb target sequence including all subjects each group

^b^ Represented as group mean for the percent base pairs (from 422 kb target sequence) with a DOC ≥1×, ≥ 10×, ≥ 20×, or ≥ 30 ×

To further ensure accuracy of variant and genotype calling, quality-based variant detection tools were employed with the following parameters: diploid organism, probability of non-reference allele ≥95% (versus sequencing error), ≥ 10-fold coverage (10×), ≥ 20% read frequency, and ≥ 30 per base quality score at the variant location. Resulting sequence variation reports were exported in variant call format (VCF) for downstream annotation.

UGT1A1*28 carrier status was separately determined by manually assessing the number of TA repeats in the NGS sequence of individual reads mapped to the promoter region (*n* = 235). Each subject’s promoter region was interpreted as the percentage of mapped reads with six TA (TA)_6_ repeats, with subject values clustering into three separate groups in a histogram (Fig. 4a). We evaluated concordance of UGT1A1*28 genotype determined by NGS with a previously reported TaqMan assay [39] in a subgroup of 81 subjects.

*CYP2D6* whole gene CNV was determined from NGS data using the bioinformatics tool CNV caller, an application within the VarSeq v1.3.4 variant annotation software (Golden Helix, Bozeman, MT), as previously described by Iacocca et al. [40]. VarSeq CNV caller identifies probable CNV carriers through coverage analysis, by normalizing the coverage across the *CYP2D6* gene for samples of interest compared to a reference control group (2 gene copies). CNV analysis was restricted to samples processed in the 24- sequencing cluster runs (*n* = 183) to minimize confounding of sample preparation on coverage. First, we determined the *CYP2D6* CNV status in 48 samples using a TaqMan CNV assay (Hs04502391_cn), from which 30 subjects with 2 *CYP2D6* copies were selected for our reference control group. CNV status for the remaining subjects was then determined using this reference group. Only subjects that were found to be positive for a CNV (deletion or duplication) were further confirmed among the remaining 135 subjects using the TaqMan CNV assay.

### Variant annotation and in silico functional assessment

Functional annotation of SNVs was carried out using ANNOVAR [41] through in silico prediction algorithms such as Combined Annotation Dependent Depletion (CADD) [42], Sorting Intolerant from Tolerant (SIFT) [43], and PolyPhen-2 [44], and variant frequency among different populations was determined utilizing large genomic databases (Reference Sequence [RefSeq], Single Nucleotide Polymorphism database build 150 [dbSNP150], 1000 Genomes, Exome Aggregation Consortium [ExAC]) obtained October 17, 2018. SNVs with a CADD Phred score (scaled) greater than 20 [42], a SIFT score of less than 0.05 [43], or a PolyPhen-2 score of greater than 0.85 [44] were considered as potentially functional variants (altering protein function), and herein defined as deleterious. Variants were classified as 1) non-synonymous (coding variants resulting in amino acid change), 2) synonymous (coding variants without amino acid changes), 3) frameshift deletion or insertion (in-del), 4) splicing (2 nucleotides within an intron-exon boundary), 5) stop gain or loss, or 6) functional intronic or promoter variants. Coding variants were further grouped by MAF reported in the ExAC database as common (MAF ≥ 5%), low frequency (1% ≤ MAF > 5%), or the combined category of rare (MAF < 1%) and novel (absent from ExAC and dbSNP build 150 databases). In silico functional assessment was restricted to protein-coding genetic variation and gain or loss of a stop codon.

### Concordance assessment

To assess concordance of clinically actionable NGS variant data, orthogonal genotyping was performed using TaqMan allelic discrimination for 39 clinically relevant SNVs including UGT1A1*28 and *CYP2D6* CNV. SNVs were chosen according to the level of evidence as defined by the Pharmacogenomics Knowledge Base or PharmGKB (http://www.pharmgkb.org/clinicalAnnotations accessed October 17, 2018) including 21 Level 1A SNVs with published prescribing recommendations for genotype-based dose adjustment or drug avoidance. Rare NGS variants were confirmed retrospectively by Sanger sequencing within 4 highly polymorphic pharmacogenes, namely *ABCB1, CYP2D6, SLCO1B1,* and *SLCO1B3*. PCR conditions and sequencing primers as well as TaqMan assay IDs are listed in Additional file 1: Table S3 and Table S4, respectively.

## Results

### Sequencing performance

Prior to alignment to the reference genome, sequencing data for all 246 subjects was assessed for read count, base quality, and GC distribution (Fig. 1 and Additional file 2: Figures S1-S3). The total number of sequenced reads per subject was dependent on sequencing cluster size, and one subject was identified to have very low read count (< 1 k reads) (Additional file 2: Figure S1). The majority of reads showed an average base quality score above 30 (Phred scale) among the 13 sequencing runs performed (Additional file 2: Figure S2). The average GC content of reads (per subject) was 45.6 ± 2.0% (mean ± SD). GC content distribution deviated greatly in 9 subjects compared to the remaining cohort (Additional file 2: Figure S3). After alignment of reads to the reference genome, we assessed coverage across the target sequence, and identified 11 subjects with greater than 20% of their target sequence ≤10× read depth, including those with high GC content. Overall, we observed a negative correlation between the low coverage and high GC content (Additional file 2: Figure S3 B). In order to avoid false negative variant calling as previously reported [38], 11 subjects with low reads and/or high GC content were removed from further analysis.

**Fig. 2:**
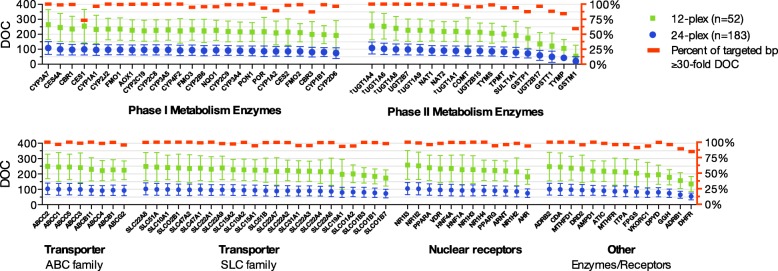
Depth-of-coverage (DOC) assessment by gene according to size of sequence cluster (*n* = 12 or 24). ^†^ For *UGT1A4, UGT1A6, UGT1A8,* and *UGT1A9*, the first exon was included to calculate DOC while shared exons were assessed only once with *UGT1A1*. Data are shown as average (±SD) median gene coverage per subject

**Fig. 3:**
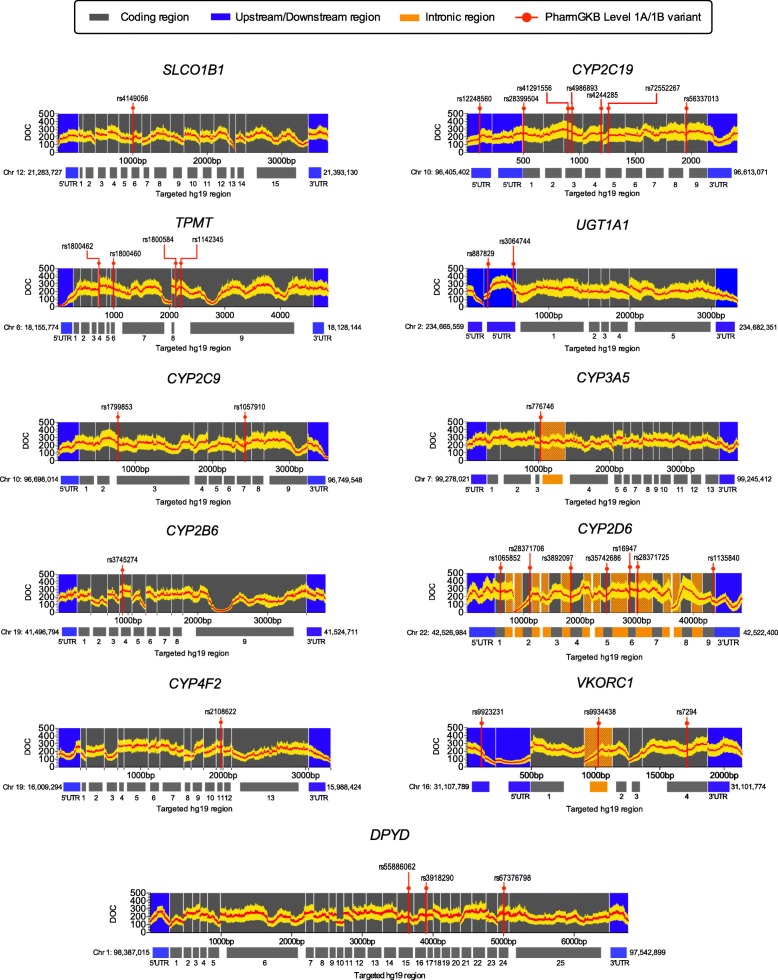
Depth-of-coverage (DOC) across the targeted sequence of 11 clinically relevant genes (*n* = 24; Sequencing Run 1 and 2). PharmGKB level 1A/1B variants (http://www.pharmgkb.org/clinicalAnnotations) are represented by rs number and genomic position by vertical lines (*red*). Data are presented as mean (±SD). PharmGKB, Pharmacogenomics Knowledge Base

Accordingly, NGS data of 235 subjects were included for subsequent coverage analysis, and assessed by sequencing cluster (*n* = 12 or 24) (Table 1). As expected, samples in the smaller cluster had a greater mean DOC per subject compared to those sequenced in the larger 24 DNA sample cluster (Table 1). Overall, the proportion of bases with a read depth ≤ 10× was very small (2–3.2%). On a gene-by-gene basis, on average, 98 of the 100 genes on our panel had a median DOC ≥50×, with ≥80% of the target region within these genes having DOC ≥30× representing deep sequencing (Fig. 2). We observed overall high coverage across clinically relevant pharmacogenes including regions of PharmGKB Level 1A/1B variants (http://www.pharmgkb.org/clinicalAnnotations) (Fig. 3). Among all genes, the glutathione S-transferase (*GST) M1* gene showed the lowest coverage per subject and large intersubject variability (min-max; 0–310×). For carboxylesterase 1 (*CES1*), there was lack of coverage for exons 12 to 14, resulting in a high proportion of targeted regions < 30×, followed by carbonyl reductase 3 (*CBR3*) (Additional file 2: Figure S4).

### Accuracy of variant detection

Genotyping with TaqMan assays was utilized to validate 39 clinically relevant variants across 215 subjects (9 variants per subject, on average) detected with NGS data (Table 2). We observed 99.9% concordance between NGS-derived and TaqMan-derived genotypes confirming heterozygous and homozygous carrier status. While we did not detect any false positive results (a variant was detected by NGS but not confirmed by TaqMan genotyping; specificity of 100%, 95% CI, 100–100%), a false negative NGS result was observed in two heterozygous carriers for *DPYD* rs67376798 and CYP2D6*10 rs1065852 (no variant detected by NGS but observed by TaqMan genotyping; sensitivity of 99.7%, 95% CI, 99.2–100%). However, subsequent assessment of individual reads revealed a variant in both subjects that was previously not called due to the low coverage in the SNV region, since the threshold for variant detection was not met (DOC ≥10x). Using Sanger sequencing, we were able to retrospectively confirm five rare coding variants that were identified by NGS in *ABCB1, CYP2D6, SLCO1B1,* and *SLCO1B3* (Additional file 1: Table S5).

**Table 2:** Concordance rate (%) between PGxSeq sequencing data compared to TaqMan-derived genotypes for clinically important SNVs as defined by PharmGKB

PharmGKB	Gene	Allele	Nucleotide change	Effect	dbSNP	Allele frequency			TaqMan			Concordanceª	FP	FN
Level of evidence					150	Study	1000G	ExAC	Patients genotyped (N)			(%)	(%)	(%)
						(*n* = 235)	EUR	EUR	WT	HET	HOM			
1A	*CYP2C19*	*17	C > T	promoter	rs12248560	0.23	0.23	NR	23	11	1	100	0	0
1A	*CYP2C19*	*2	G > A	p.P227P	rs4244285	0.14	0.15	0.15	25	11	0	100	0	0
1A	*CYP2C19*	*3	G > A	p.Y212X	rs4986893	ND	0	1.80E-04	50	0	0	100	0	NA
1A	*CYP2C9*	*2	C > T	p.R144C	rs1799853	0.13	0.12	0.13	72	23	3	100	0	0
1A	*CYP2C9*	*3	A > C	p.I359L	rs1057910	0.06	0.06	0.07	87	13	0	100	0	0
1A	*CYP2D6*	*10	C > T	p.P34P	rs1065852	0.21	0.2	0.25	30	19	3	98.1	0	1.9
1A	*CYP2D6*	*4	G > A	splice	rs3892097	0.19	0.19	0.17	52	26	6	100	0	0
1A	*CYP2D6*	*3A	A > del	p.R208fs	rs35742686	0.03	0.02	0.02	50	2	0	100	0	0
1A	*CYP2D6*	*41	G > A	intronic	rs28371725	0.12	0.09	0.09	38	14	0	100	0	0
1A	*CYP3A5*	*3	A > G	splice	rs776746	0.93	0.95	NR	1	9	27	100	0	0
1A	*CYP4F2*	*3	C > T	p.V433 M	rs2108622	0.30	0.27	0.29	23	17	0	100	0	0
1A	*DPYD*	*13	T > G	p.I560S	rs55886062	2.1E-03	1.3E-03	6.18E-04	97	1	0	100	0	0
1A	*DPYD*	*2A	G > A	splice	rs3918290	0.02	0.01	0.01	100	1	0	100	0	0
1A	*DPYD*		A > T	p.D949V	rs67376798	0.02	2.2E-03	4.09E-03	80	8	0	98.9	0	1.1
1A	*SLCO1B1*	*5	T > C	p.V174A	rs4149056	0.18	0.17	0.16	67	31	4	100	0	0
1A	*TPMT*	*2	G > C	p.A80P	rs1800462	2.1E-03	6.00E-03	1.97E-03	51	1	0	100	0	0
1A	*TPMT*	*3B	G > A	p.A154T	rs1800460	0.04	0.03	0.04	41	10	0	100	0	0
1A	*TPMT*	*4	G > A	splice	rs1800584	ND	NR	3.01E-05	51	0	0	100	0	NA
1A	*TPMT*	*3C	A > G	p.Y240C	rs1142345	0.04	0.03	0.04	41	10	0	100	0	0
1A	*UGT1A1*	*28	(TA)_6_ > (TA)_7_	promoter	rs3064744	0.32	0.29	NR	36	40	5	100	0	0
1A	*VKORC1*		G > A	intergenic	rs9923231	0.40	0.40	NR	19	15	6	100	0	0
1B	*CYP2B6*	*9	G > T	p.Q172H	rs3745274	0.21	0.23	0.24	22	9	0	100	0	0
2A	*ABCB1*		C > T	p.I1145I	rs1045642	0.48	0.47	0.47	18	61	26	100	0	0
2A	*CYP2D6*	*9	AAG > del	p.K281del	rs5030656	0.02	0.02	0.03	48	4	0	100	0	0
2A	*SLCO1B1*	*1B	A > G	p.N130D	rs2306283	0.41	0.40	0.41	40	46	15	100	0	0
2B	*ABCG2*		C > A	p.Q141K	rs2231142	0.12	0.10	0.10	87	20	1	100	0	0
3	*ABCC2*		G > A	p.V417I	rs2273697	0.19	0.20	0.20	45	19	4	100	0	0
3	*ABCG2*		G > A	p.V12 M	rs2231137	0.04	0.06	0.05	81	9	0	100	0	0
3	*CYP2B6*		C > T	p.R487C	rs3211371	0.12	0.10	0.12	23	8	0	100	0	0
3	*CYP3A4*	*22	C > T	intronic	rs35599367	0.05	0.05	NR	36	8	0	100	0	0
3	*DPYD*	*HapB3*	G > A	p.E412E	rs56038477	0.03	0.02	0.02	73	11	0	100	0	0
3	*SLCO2B1*		G > A	p.R290Q	rs12422149	0.06	0.10	0.11	62	6	0	100	0	0

ª Percentage of total tested DNA samples with NGS-determined genotypes concordant with TaqMan results. False positive was defined as TaqMan determined “homozygous wildtype” and NGS determined “variant carrier”. False negative was defined as TaqMan determined “variant carrier” and NGS determined “homozygous wildtype”. PharmGKB definition for levels of evidence can be found at https://www.pharmgkb.org/page/clinAnnLevels. Nucleotide change presented as the change on the coding strand. Abbreviations: *dbSNP 150* Single Nucleotide Polymorphism database build 150, *ExAC* Exome Aggregation Consortium European dataset, *FP* false positive, *FN* false negative, *HET* heterozygous genotype, *HOM* homozygous genotype, *ND* not detected in our patient database, *NA* not applicable as no variant carriers were found, *NR*, not reported in, 1000G EUR, or ExAC database, *1000G EUR* 1000 Genomes Project European dataset, *PharmGKB* Pharmacogenomics Knowledge Base

**Fig. 4:**
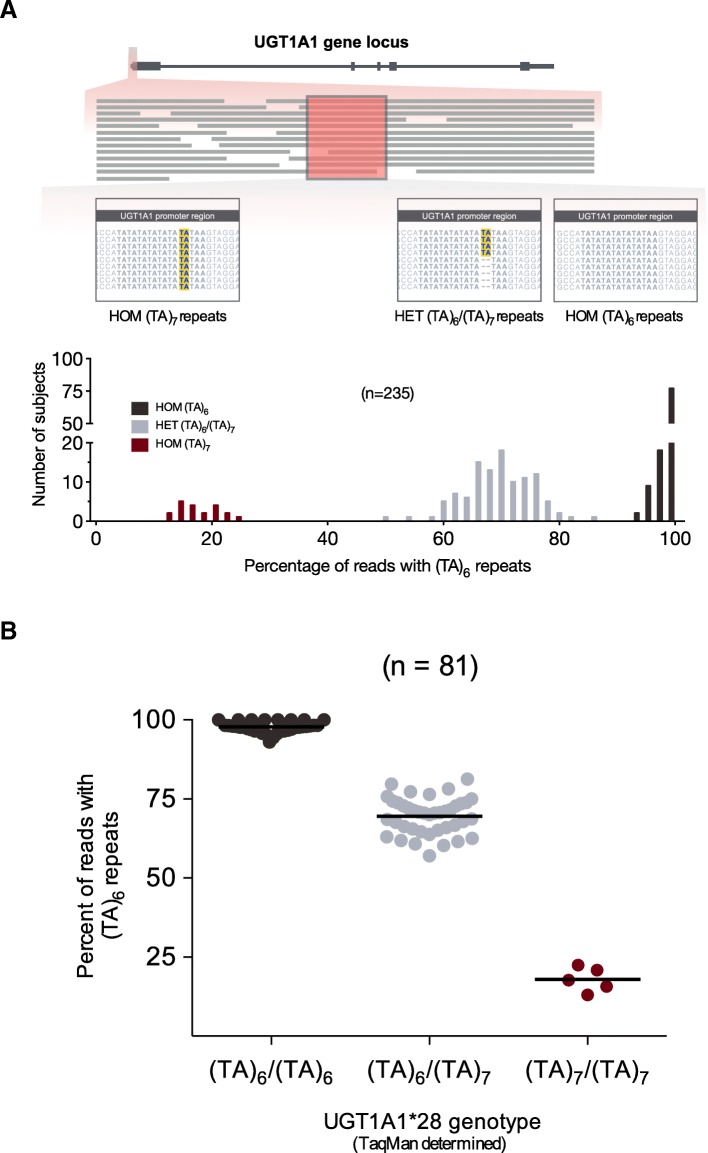
Determination of UGT1A1*28 (TA)_7_ promoter repeat by next-generation sequencing (NGS). **a** Schematics of manual assessment of aligned reads within the *UGT1A1* promoter region, and multimodal frequency distribution pattern of UGT1A1*28 genotype. **b** Confirmation of NGS determined UGT1A1*28 genotype by TaqMan assay

UGT1A1*28 polymorphism detection using NGS data was carried out manually in 235 individuals. Each subject’s promoter region was expressed as the percentage of reads with six TA repeats, which clustered into 3 separate groups according to their frequency distribution (Fig. 4a), and UGT1A1*28 genotype (*1/*1, *1/*28, *28/*28) confirmed by TaqMan genotyping in a subset of 81 subjects (Fig. 4b; Table 2).

**Fig. 5:**
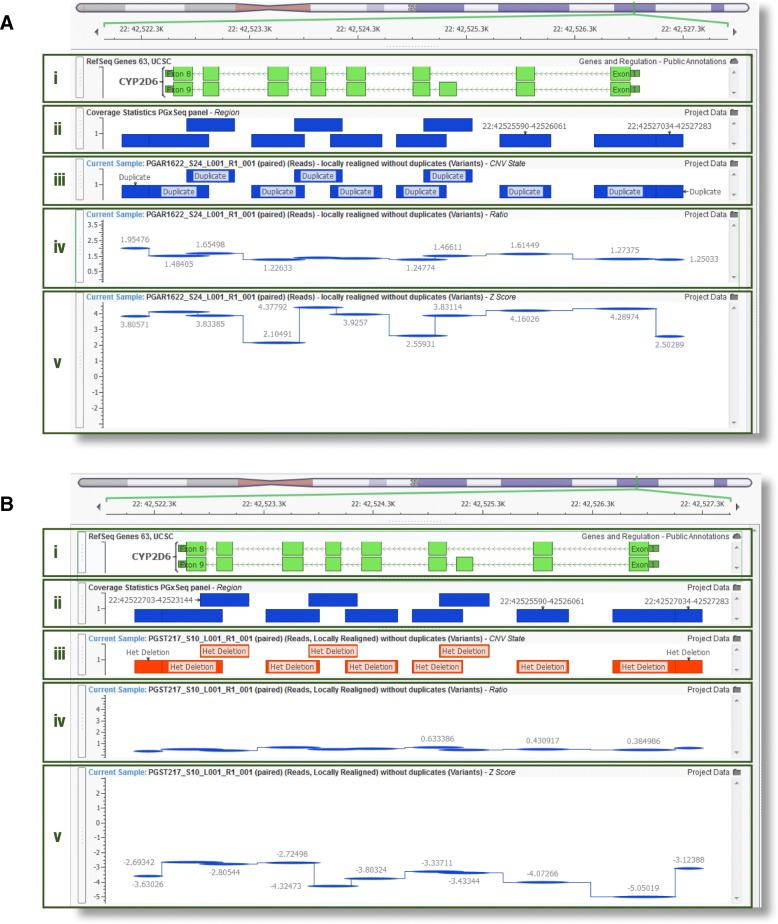
Next-generation sequencing (NGS)-based detection of *CYP2D6* copy number variation. **a** Representative NGS output for a duplication of the whole *CYP2D6* gene (Subject PGAR1622 with CYP2D6*1/*1 genotype; refer to Table 2 for more detail). **b** Representative NGS output for a heterozygous deletion of the whole *CYP2D6* gene (Subject PGST217 with CYP2D6*1/*5 genotype; refer to Table 2 for more detail). Different regions of the output are as follows: (i) Exon map of the *CYP2D6* gene. (ii) PGxSeq probe target regions. (iii) Called CNV state per probe target region, determined by ratio and z-score metrics. (iv) Normalized ratio metric computed for each NGS probe target region in *CYP2D6*; sample coverage compared to reference controls (*N* = 30). (v) Z-score metric: number of standard deviations the depth of coverage is from the reference control mean coverage

*CYP2D6* whole gene CNV analysis was performed in all subjects processed in the 24 sample sequencing cluster (*n* = 183). A representative sample output for a subject with a *CYP2D6* duplication and deletion is shown in Fig. 5. We detected a gene deletion (heterozygous form) and duplication in 2.5 and 3.3% of subjects, respectively, which were confirmed by TaqMan CNV assay (Table 3**)**. Notably, *CYP2D6* genotype revealed duplications of *4 and/or *41 alleles in three out of 6 patients resulting in a predicted intermediate metabolizer phenotype for CYP2D6. In addition, gene deletion occurred in combination with *3 and *4 alleles, resulting in a predicted poor metabolizer status in four out of 9 patients.

**Table 3:** NGS-based detection of *CYP2D6* whole gene copy number variation (CNV) in 183 subjects. For more detail on the detection, refer to Fig. 5

*Subject ID*	*CYP2D6* copy number detection					CYP2D6 genotype	Phenotype prediction
	NGS			TaqMan^a^			
	Coverage ratio	Z-score	Gene copy number	Ratio	Gene copy number		
*PGAR844*	0.55	−3.16	1	0.44	1	*1/*5	IM
*PGAR867*	0.51	−3.36	1	0.44	1	*1/*5	IM
*PGON198*	0.50	−2.96	1	0.53	1	*4/*5	PM
*PGST66*	0.43	−3.54	1	0.45	1	*3/*5	PM
*PGST140*	0.48	−3.39	1	0.43	1	*1/*5	IM
*PGST217*	0.45	−3.55	1	0.45	1	*1/*5	IM
*PGST52*	0.54	−2.74	1	0.45	1	*1/*5	IM
*PGAR1070*	0.47	−3.33	1	0.48	1	*4/*5	PM
*PGAR1132*	0.46	−3.79	1	0.49	1	*4/*5	PM
*PGAR1622*	1.44	3.59	> 2	1.40	> 2	*1/*1	UM
*PGON142*	1.30	3.20	> 2	1.48	> 2	*1/*1	UM
*PGON287*	1.62	4.04	> 2	1.90	> 2	*41/*4	IM
*PGST38*	1.32	1.91	> 2	1.38	> 2	*1/*4	IM
*PGON194*	1.29	2.25	> 2	1.98	> 2	*1/*4	IM
*PGST223*	1.60	3.25	> 2	1.86	> 2	*1/*1	UM

*PM* poor metabolizer, *IM* intermediate metabolizer, *UM* ultrarapid metabolizer

^a^ Validation by TaqMan CNV assay in subjects that were identified with CNV (*n* = 15), and 48 subjects initially characterized to select a reference control group (*n* = 30)

### Analysis of variants in pharmacogenes

**Fig. 6:**
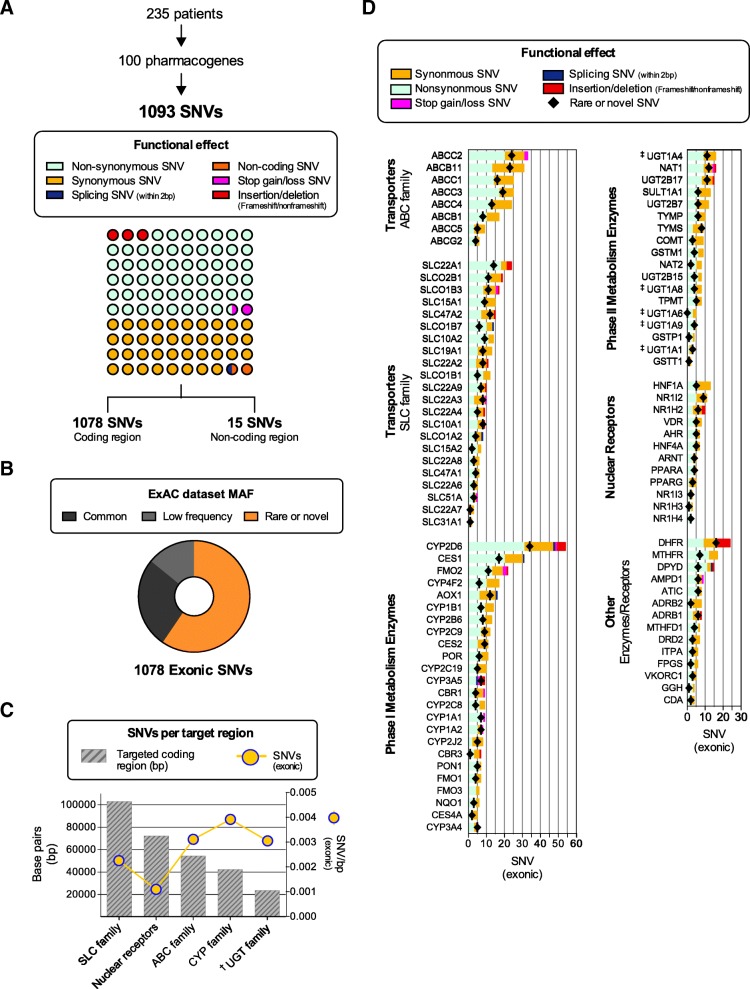
Assessment of genetic variation determined by PGxSeq in 235 subjects. According to functional effect (**a**), allele frequency reported in ExAC database (**b**), number of exonic variants per target region (**c**), and gene (**d**). ^‡^ For *UGT1A4, UGT1A6, UGT1A8,* and *UGT1A9,* only SNVs located within the first exon were counted while shared exons were assessed only once with *UGT1A1*. ExAC, Exome Aggregation Consortium; MAF, minor allele frequency

Genetic variation was assessed in 235 Caucasian study subjects (Additional file 1: Table S6), and SNVs presented in Fig. 6 according to functional effect, number of variants per gene, and reported MAF in ExAC (if exonic), the latter capturing NGS exome data of 60,706 individuals [45]. A total of 1093 unique SNVs were identified, consisting of 605 non-synonymous (55.4%), 417 synonymous (38.1%), 7 splice-site (0.6%), 14 stop gain or loss (1.4%), and 35 insertion-deletions (18 frameshift, 17 non-frameshift; 3.2%), as well as 15 known non-coding SNVs (1.4%) (Fig. 6a). The majority of variants (72%) were only present in heterozygous form. Among exonic variants (Fig. 6b), 26.3% of SNVs were common (ExAC MAF > 5%), 14.2% occurred at a low frequency (ExAC MAF ≥1 and ≤ 5), whereas 59.5% were either rare or novel (ExAC MAF < 1% or absent from ExAC or dbSNP150). MAFs in this study largely mirrored those reported in much larger data sets of subjects with European descent (ExAC, 1000G) (Additional file 2: Figure S5). According to gene family or drug-related function, the *CYP* gene families had the most variants per targeted base pairs, followed by the *ABC* and *UGT* family, then *SLC* family, while nuclear receptors were the least variable (Fig. 6c). Individually, among Phase I enzymes, *CYP2D6* had the highest total number of exonic SNVs (54) and the highest number of rare or novel variants from our gene panel (Fig. 6d**)**, whereas *UGT1A4* and N-acetyltransferase 1 (*NAT1)* had the most SNVs among Phase II enzymes. Within the *SLC* family, organic cation transporter 1 (*SLC22A1*) showed the highest number of SNVs as well as rare or novel SNVs among all *SLC* genes sequenced. Among transporter genes of the *ABC* family, *ABCC2* had the highest number of the SNVs with 33 variants. No variants were detected for *CYP3A7* and *SLC51B*, despite adequate coverage achieved across both coding sequences.

### In silico assessment of variants in pharmacogenes

**Fig. 7:**
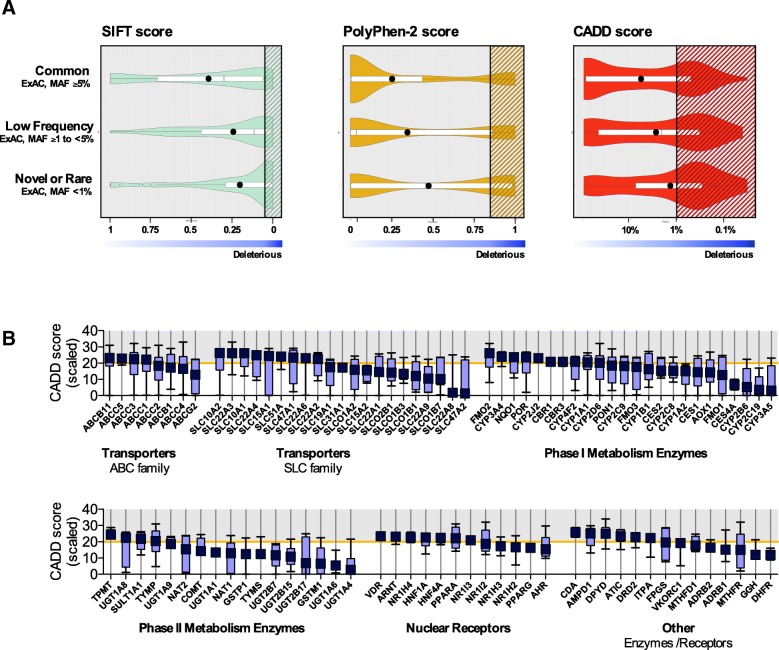
In silico assessment of non-synonymous variation in pharmacogenes identified by PGxSeq (*N* = 235). **a** Frequency distribution of variants according to SIFT, PolyPhen-2, and CADD scores separated by minor allele frequency reported in the ExAC database. Shaded regions represent the proportion of potentially functional variants (or deleterious), defined as a scaled CADD score > 20 [42], a SIFT score < 0.05 [43], or a PolyPhen-2 score > 0.85 [44]. **b** Box and whisker plots of scaled CADD scores separated by gene; whiskers represent 10-90th percentile with purple symbol (■) representing the median. ABC, ATP binding cassette; CADD, Combined Annotation Dependent Depletion; ExAC, Exome Aggregation Consortium; MAF, minor allele frequency; SIFT, Sorting Intolerant from Tolerant; SLC, solute carrier

Potential functional effects of the identified non- synonymous variants were assessed with CADD, PolyPhen- 2, and SIFT. Our results showed marked differences between the prediction scores derived from these algorithms (Fig. 7a). However, the proportion of rare (MAF < 1%) or novel variants that were categorized as possibly deleterious was greater than the proportion of common (≥ 5%) or low frequency (≥ 1–5%) variants for all 3 tools (CADD: *p* = 0.0002, PolyPhen-2: *p* = < 0.0001, and SIFT: *p* = 0.0002) (Additional file 2: Figure S6). The majority of pharmacogenes (96 out of 100) harbored at least one variant with a CADD score > 20 (median, 5) (Fig. 7b). On average, 14.8% (9.3–21.3%, min-max) of the coding (exonic) variants detected across the 100 pharmacogenes per subject were predicted as deleterious (CADD score > 20). Although the majority of these variants were observed in heterozygous form (Additional file 2: Figure S7), all 235 subjects had ≥1 deleterious variant(s) in the homozygous form, with a median of 4 (1–12, min-max) SNVs per subject. Finally, we assessed prediction scores among 12 CYP genes that account for the majority of reported drug oxidation reactions (Additional file 1: Figure S8); on average, 11% of SNVs (10 - 90th percentile, 4.1–20.0%) with a CADD > 20 among individuals were located within these genes.

### Variation in genes of clinical relevance

Among 11 clinically relevant genes for which prescribing guidelines for specific gene-drug combinations have been published (cpicpgx.org/genes-drugs/), there are 31 PharmGKB Level 1A and 1B variants categorized as having strong evidence for influencing drug efficacy/response and/or prescribing recommendations (Fig. 3). We identified 24 out of these 31 variants in our cohort, with 183 patients (78%) harbouring at least one PharmGKB level 1A/1B homozygous variant (Additional file 2: Figure S9).

## Discussion

As genotype-guided pharmacotherapies advance into the clinical setting, targeted NGS technologies provide great utility by simultaneously detecting common as well as rare genetic variation of potential relevance to adverse or desired drug response in patients. In this study, we established and validated a comprehensive targeted PGxSeq exome panel for most clinically important pharmacogenetic loci. Our findings demonstrate excellent concordance for the detection of clinically relevant variants compared to standard pharmacogenetic assays including the UGT1A1*28 promoter (TA)_7_ TAA repeat and *CYP2D6* copy number variation. Moreover, adequate read depth along the target regions and a strong correlation of allelic frequencies for rare and novel variants in this population compared to larger genetic datasets suggests accurate and reliable results, while confirming the high prevalence of such potentially functional variation within pharmacogenes.

Compared to traditional genotyping or sequencing strategies, the applied targeted exome sequencing strategy enabled accurate genotyping for common, previously established functional variation across exonic and intergenic regions in clinically important pharmacogenes as well as the comprehensive discovery of novel rare SNVs with fast and adequate performance. Available bioinformatics tools further allowed customized utilization of sequencing data at a small or large scale, i.e. the assessment of individual genotypes and genes of interest or a more exhaustive pharmacogenetic analysis. Importantly, the majority of patients (78%) harbored one or more homozygous PharmGKB Level 1A or 1B variant(s) with recommendations to adjust dose or for alternative therapies confirming recent findings from the eMerge-PGx study comprising extensive sequencing data from 5000 patients for 82 pharmacogenes [46]. Moreover, 60% of the observed SNVs were rare (536 variants; 2.3 per patient) or novel (105 variants; 4.4 per 10 patients), the latter absent in more than 60,000 individuals [45]; a similar frequency of 73% has been previously reported in a whole-genome sequencing study of 231 pharmacogenes [20]. Accordingly, a significant portion of novel variation will likely be missed when utilizing more cost-effective, array-based genotyping platforms such as DMET+ (1936 SNVs in 231 pharmacogenes; Affymetrix, CA, USA) or the genome-wide Infinium Global Screening Array-24 (665,608 SNVs; Illumina, CA, USA). Moreover, the capacity of assessing CNV in pharmacogenes is an additional advantage of this NGS panel. Overall, the multitude of other, newly discovered candidate variants among pharmacogenes in this study highlights the need for comprehensive sequencing approaches to determine the likely more complex genotype of a patient, while high-throughput experimental strategies are warranted to screen and confirm effects of previously unreported genetic variation on protein activity.

While NGS is thought to be best suited for the detection of SNV, most recent reports highlight its utility for the identification of genomic structural variants as demonstrated for *GSTs* [27], the LDL receptor (*LDLR*) [40], the PCSK9 enzyme (*PCSK9*) [47], and various genes underlying retinal dystrophies [48], among others. Our findings demonstrate that a read-depth based approach can be successfully applied for the identification of CNV in *CYP2D6*, a gene notorious for its complex genomic architecture and pseudogene homology [30, 49]. *CYP2D6* gene deletion (*5 allele) and multiplication are commonly observed among various ethnicities (2–3% in Caucasians) [11], resulting in reduced (or lack of) and increased enzymatic activity, respectively. Previously, *CYP2D6* CNV has been assessed in 61 adult [33] as well as 98 pediatric patients [50] utilizing whole-genome sequencing data, while targeted NGS pharmacogene panels have not reported such results [27, 28]. While previous approaches evaluating whole-genome sequences have failed to predict *CYP2D6* CNV in several subjects [33, 50], we were able to confirm concordance in all assessed patients using the bioinformatics tool VarSeq CNV caller. Accuracy of these results is further supported by frequencies observed in this study that are in close agreement with the literature with 3.3% for *CYP2D6* duplication (*1xN and *4xN), and 2.5% for *CYP2D6* deletion (*1/*5, *3/*5 and *4/*5). Our findings clearly indicate that information regarding *CYP2D6* genotype and CNV is critical for accurate CYP2D6 phenotype prediction, exemplified by duplication of non-functional alleles such as *4. Known to metabolize about 25% of commonly prescribed drugs [51], *CYP2D6* genotype is implicated as a pharmacogenomic biomarker in drug labelling in about 25% of medications currently listed by the FDA (http://www.fda.gov/Drugs/ScienceResearch/ucm572698.htm), and genotype-based prescribing guidelines have been previously published for tamoxifen [11], codeine [52], and tricyclic antidepressants [53].

To our knowledge, this is the first study reporting the utility of NGS data to identify UGT1A1*28 allele status. However, for this purpose, manual assessment of the (TA)n repeat in sequencing reads within the *UGT1A1* promoter region was required for each subject. Additional bioinformatics tools are warranted to automate variant calling of UGT1A1*28 to enable high-throughput analysis in large patient numbers. UGT1A1*28 has been reported to lower glucuronidation rate of the active metabolite of irinotecan, SN-38, likely associated with higher toxicity [54, 55], and is currently part of prescribing guidelines for atazanavir [14].

A significant number of pharmacogenetic variants detected in our validation cohort was either rare or novel (60%), and more than half (55%) resulted in amino acid changes, supporting previous observations in larger datasets [17, 46, 56, 57]. While the proportion of SNVs with predicted effects on protein function differed among applied in silico tools, differences in scoring have been previously observed and are not surprising given the way these algorithms were derived [27]. SIFT leverages the evolutionary conservation of amino acids [43], PolyPhen-2 uses pathogenicity information [44], while CADD is the most recent algorithm integrating conservation metrics, regulatory information, and protein-level effect among others [42]. Moreover, a higher false negative rate may apply for predicting rare gain-of-function compared to loss-of-function variants using SIFT and PolyPhen [58], while algorithms such as CADD may be more comparable [59]. A recent study suggests that the in silico algorithms used here predict altered enzymatic or transporter function with about 80% accuracy compared to in vitro assessment [27]. Among 207 to 275 possibly deleterious variants predicted in this study, rare or novel SNVs were more likely to have functional effects than common or low frequency variants (Additional file 2: Figure S6), and accounted for 41–51% of all deleterious SNVs. These findings are similar to a recent report evaluating NGS data from thousands of individuals in 146 pharmacogenes, where 30 to 40% of rare variation was predicted to be functional [17]. Moreover, we found that nearly all patients (221 of 235) carried at least one deleterious allele (CADD score > 20) in 12 *CYP* genes with key roles in drug metabolism [56, 60]; these potentially clinically relevant findings need to be followed up.

Genetic profiling using any short-fragment sequencing platform is a widely recognized challenge for NGS of pharmacogenes [30, 61], and requires sufficient representation of mapped sequenced reads in the region of interest to ensure accuracy. As expected, many members of the *CYP*, *SULT* and *UGT* gene families were reported as harboring 250-bp sequence fragments that map to more than one place in the genome due to their sequence similarity, with regions that are up to 100% identical (i.e. pseudogenes) predicted of being the most problematic [29]. An estimated 1.8% of our 422 kb target sequence (69 exons in 19 genes) was found to be susceptible to potential mismapping. Although our hybridization-based enrichment strategy achieved a median read coverage above 50x for most genes (98 of 100), the results also indicate that the median or mean value alone may not always correctly indicate evenly sufficient read coverage across the targeted region. Specifically, for *CES1* the average median per subject was DOC ≥100x was observed, however 30.2% of its targeted bases (Exon 12–14) showed a DOC <30x indicating areas prone to higher false negative rates (Fig. 2, Additional file 2: Figure S3); a 95–100% sequence similarity has been previously reported for *CES1* exons 12–14 [29]. Accordingly, high homology regions may benefit from longer capture probes for hybridization-based target enrichment to ensure appropriate capture and/or sequence read mapping. Moreover, DOC for *GSTM1* were the lowest among all genes of our panel. A previous report in a Korean population sample showed individuals with *GST* gene deletion (GST*0) lacked coverage when assessed with NGS, while the number of gene copies correlated the mean number of sequenced read depth [27]. Deletions of *GSTs* are also prevalent among Europeans (MAF ~ 0.5 [62]), and we noted 57 and 17% of our study group had near zero coverage for *GSTM1* and *GSTT1*, respectively, likely representing GST*0 carrier status (Additional file 2: Figure S10). Our findings highlight the need for monitoring targeted regions for low sequence coverage, absent data or ambiguous calls to reduce false negative or positive findings by defining test panel limitations in agreement with current clinical laboratory standards for NGS [63].

While we show the potential application of targeted exome sequencing as a comprehensive pharmacogenetic profiling tool, there are some limitations. Validation of concordance was limited to variants in 39 loci in 16 genes in our relatively small, mostly Caucasian sample, in contrast to previously reported multi-center studies that assessed hundreds of SNVs in larger populations [27, 28] including commercially available DNA control samples [27, 63]. However, despite the small sample size, the herein observed variation compared well to findings from larger data sets. Moreover, our gene panel is largely restricted to pharmacogenes of relevance to drug disposition, while a recent report indicates the increasing relevance of drug target genes [64]. Lastly, in contrast to whole-genome sequencing, our targeted exome panel is unable to detect pharmacogenomic variants in 3′- and 5′-untranslated as well as intronic regions that may be of relevance.

## Conclusions

Next-generation sequencing platforms are starting to impact upon many clinical fields, especially cancer and pediatrics. Bringing these technologies to clinical pharmacogenetics represents a timely and logical convergence, especially given the history of applied genetic concepts and molecular methods within the discipline. Through comprehensive validation of performance and accuracy, results from our study and others demonstrate the utility of targeted exome sequencing panels as sensitive and reliable sequencing platforms for pharmacogenes, including *CYP2D6* CNV [27, 28]. But despite the relative ease of the sequencing process, the time and effort required for post-sequencing computational and bioinformatics data analyses are significant due to the technical and interpretive complexity of NGS and the biology of some pharmacogenetic gene targets. Moreover, as new variants are discovered using these high-throughput detection methods, the need for standards in attributing pathogenicity together with development of tools for high-throughput functional assessment and clinical validation are required before implementing findings to aid therapeutic decision-making.

## Additional files

Additional file 1: **Table S1.** Genomic coordinates of PGxSeq capture probe design. **Table S2.** Known promoter and intronic SNVs targeted in PGxSeq panel. **Table S3.** Polymerase chain reaction conditions and primers used for Sanger sequencing of rare NGS variants. **Table S4.** TaqMan assay ID for the validated clinically relevant SNVs. **Table S5.** Concordance of rare variation in select genes by Sanger sequencing. **Table S6.** Characterization of unique variants identified by PGxSeq in 235 Caucasian subjects. (XLSX 298 kb)

Additional file 2: **Figure S1.** Mean (±SD) read count per subject (duplicates removed) stratified by sequencing cluster (*n* = 246). **Figure S2.** Number of reads versus mean base quality score (Phred scale) per read for all sequencing runs (*n* = 246). **Figure S3.** Assessment of guanine and cytosine [GC] content within sequencing reads (*n* = 246). Histogram of the average percent GC content across total reads (A). Relationship between subjects average GC content and coverage (B). **Figure S4.** Mean (±SD) depth of coverage (DOC) across the targeted sequence for CES1 and CBR1 showing the inaccessible target regions. **Figure S5.** Study minor allele frequencies (MAF) in relation to the reported MAF in 1000 Genomes Project (1000G) and Exome Aggregation Consortium (ExAC) datasets. **Figure S6.** In silico functional prediction scores for genetic variants identified among 235 subjects. Rare or novel variations had a greater proportion of possibly deleterious prediction scores for all three algorithms (SIFT, Polyphen-2 and CADD). **Figure S7.** Zygosity of the potentially deleterious variants (CADD scaled score greater than 20) per subject (*n* = 235), showing there were more heterozygous compared to homozygous variants per subject. **Figure S8.** Single nucleotide variants (SNV) per subject (*n* = 235) found in cytochrome P450 (CYP) enzymes (CYP1A1, CYP1A2, CYP1B1, CYP2B6, CYP2C19, CYP2C8, CYP2C9, CYP2D6, CYP2J2, CYP3A4, CYP3A5, and CYP4F2) that are potentially deleterious variants (CADD scaled score greater than 20) separated by zygosity. **Figure S9.** Number of Pharmacogenomics Knowledge Base (PharmGKB) “Level 1A/1B” variants (categorized as having strong supporting evidence for affecting drug efficacy/response and/or specific prescribing recommendations https://www.pharmgkb.org/clinicalAnnotations) found in 235 subjects separated by zygosity. **Figure S10.** Histogram of the GSTM1 and GSTT1 gene coverage as a fraction total subject coverage in 235 subjects. (PDF 2439 kb)

## Abbreviations

1000G: 1000 Genomes Project
ABC: ATP binding cassette
ADME: Absorption, distribution, metabolism, excretion
CADD: Combined Annotation Dependent Depletion
CBR3: Carbonyl reductase 3
CES1: Carboxylesterase 1
CNV: Copy number variation
CPIC: Clinical Pharmacogenetics Implementation Consortium
CYP: Cytochrome P450
dbSNP150: Single nucleotide polymorphism database build 150
DOC: Depth-of-coverage
ESP: Exome sequencing project
GC: Guanine and cytosine
gDNA: Genomic DNA
GST: Glutathione S-transferase
Indels: Insertion-deletion
LDLR: LDL receptor
MAF: Minor allele frequency
NAT1: N-acetyltransferase 1
NGS: Next-generation sequencing
PCR: Polymerase chain reaction
PharmGKB: Pharmacogenomics knowledge base
RefSeq: Reference Sequence
SIFT: Sorting intolerant from tolerant
SLC: Solute carrier
SLCO: Solute carrier organic anion transporter
SNVs: Single nucleotide variants
UCSC: University of California Santa Cruz
UGT: UDP glucuronosyltransferase
UTR: Untranslated region
VCF: Variant call format
