# Supplementary material for: Targeted next generation sequencing as a tool for precision medicine
Source: BMC Med Genomics. 2019 Jun 3;12:81. doi: 10.1186/s12920-019-0527-2 (PMC6547602; doi:10.1186/s12920-019-0527-2)

Figure S1. Mean (±SD) read count per subject (duplicates removed) stratified by sequencing cluster (n=246).

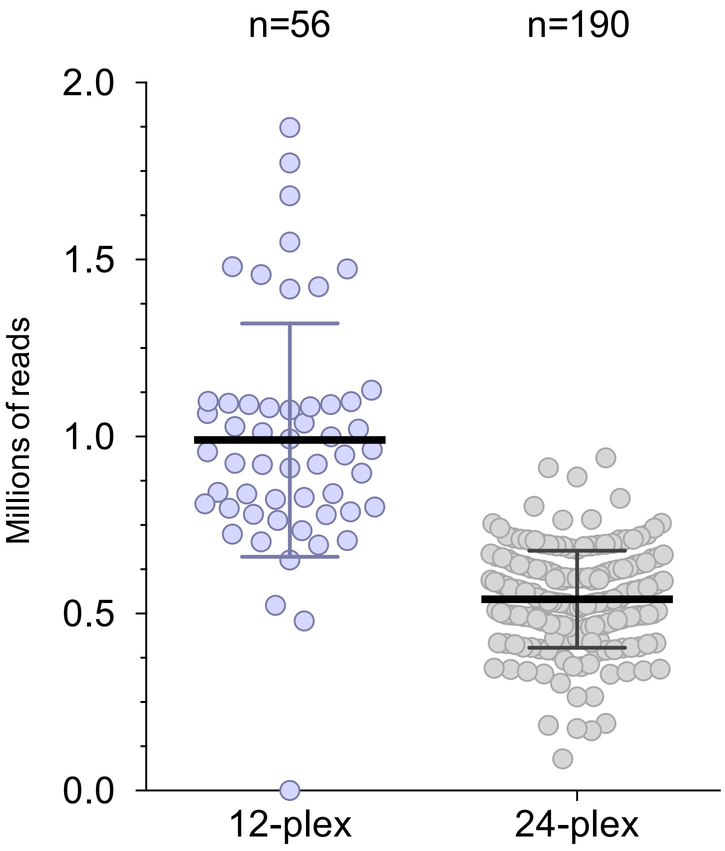

Figure S2. Number of reads versus mean base quality score (Phred scale) per read for all sequencing runs (n=246).

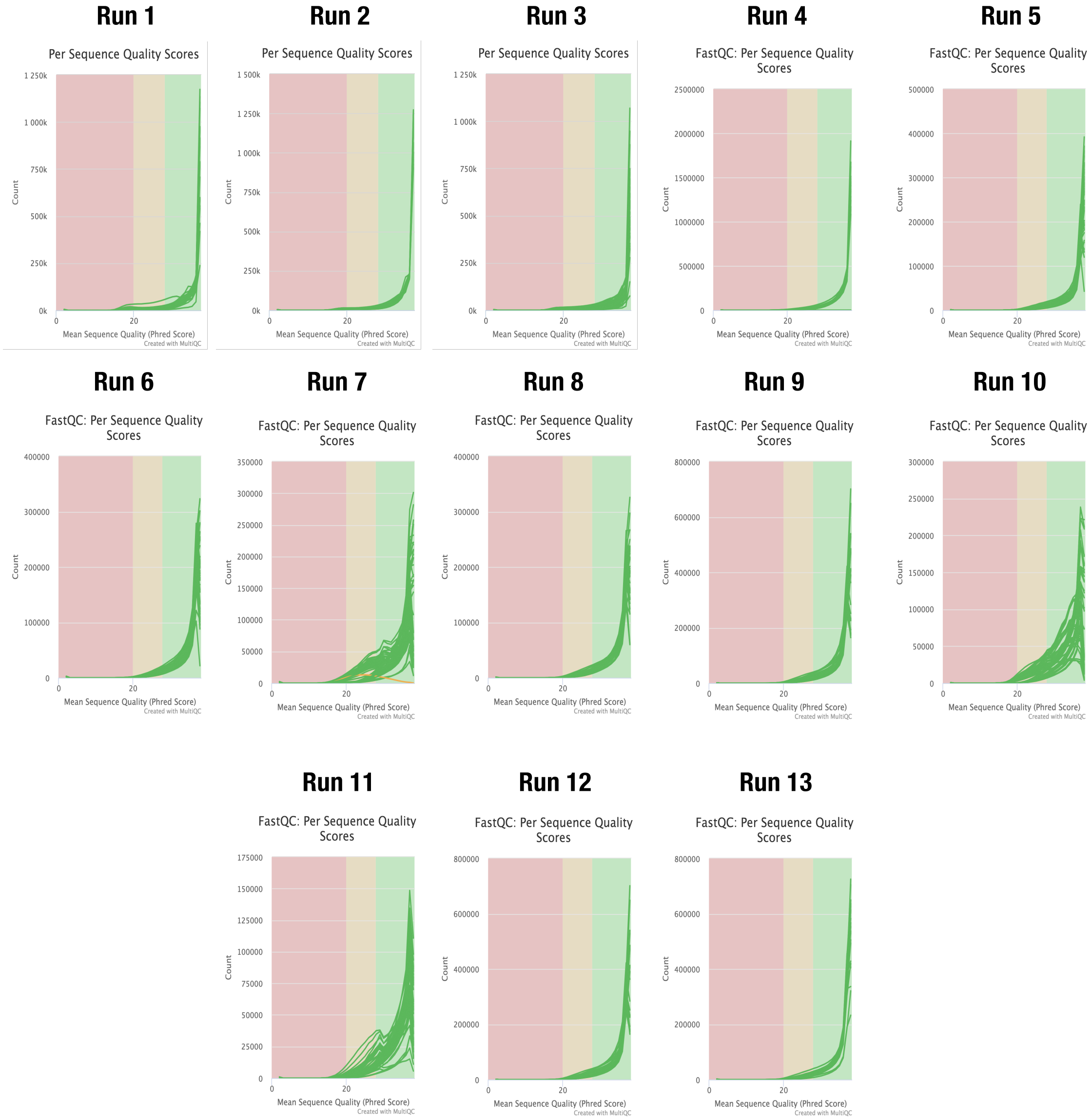

Figure S3. Assessment of guanine and cytosine [GC] content within sequencing reads (n=246). Histogram of the average percent GC content across total reads (A). Relationship between subjects average GC content and coverage (B). Abbreviation: DOC, depth of coverage

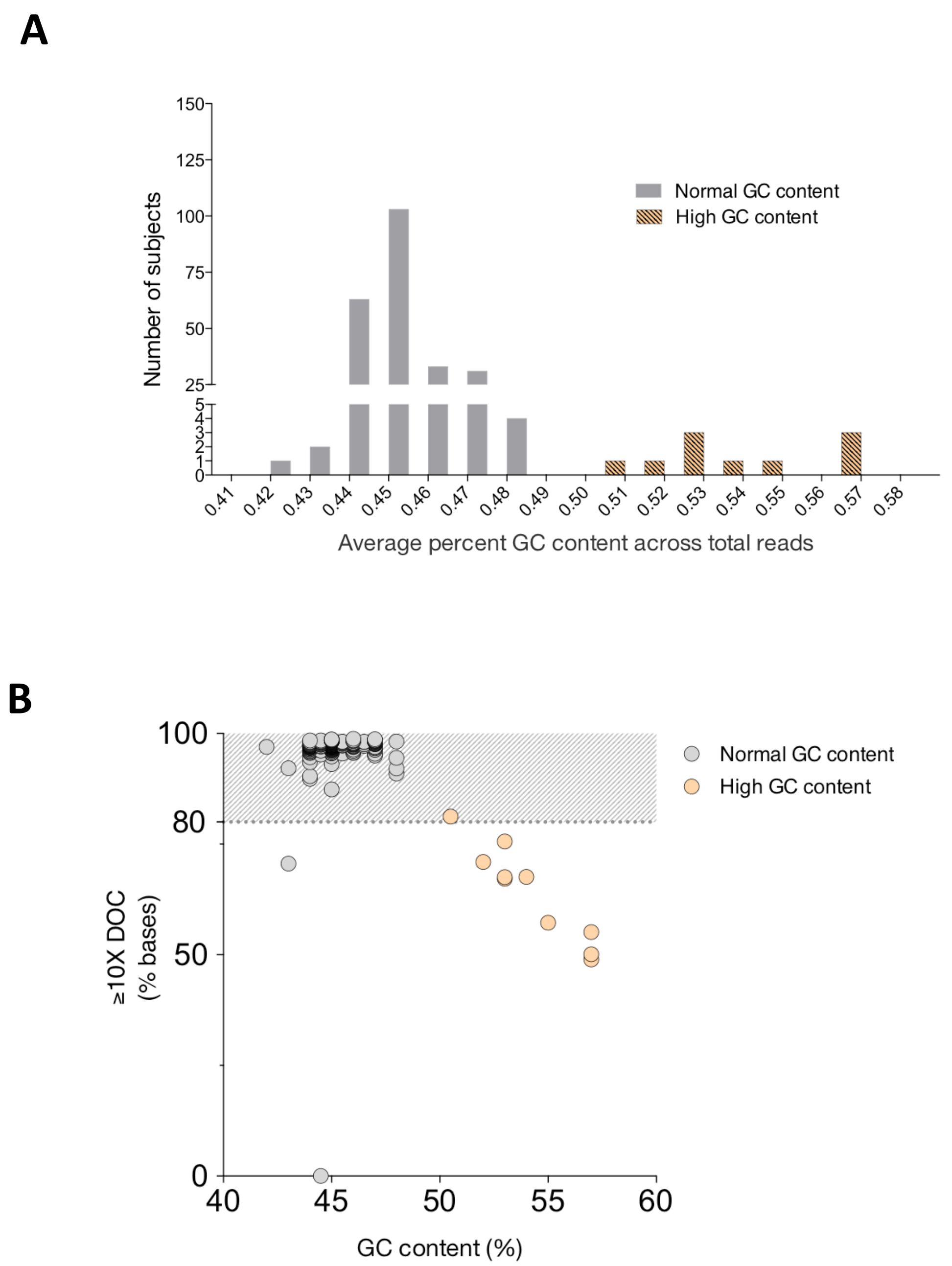

Figure S4. Mean (±SD) depth of coverage (DOC) across the targeted sequence for *CES1* and *CBR1* showing the inaccessible target regions.

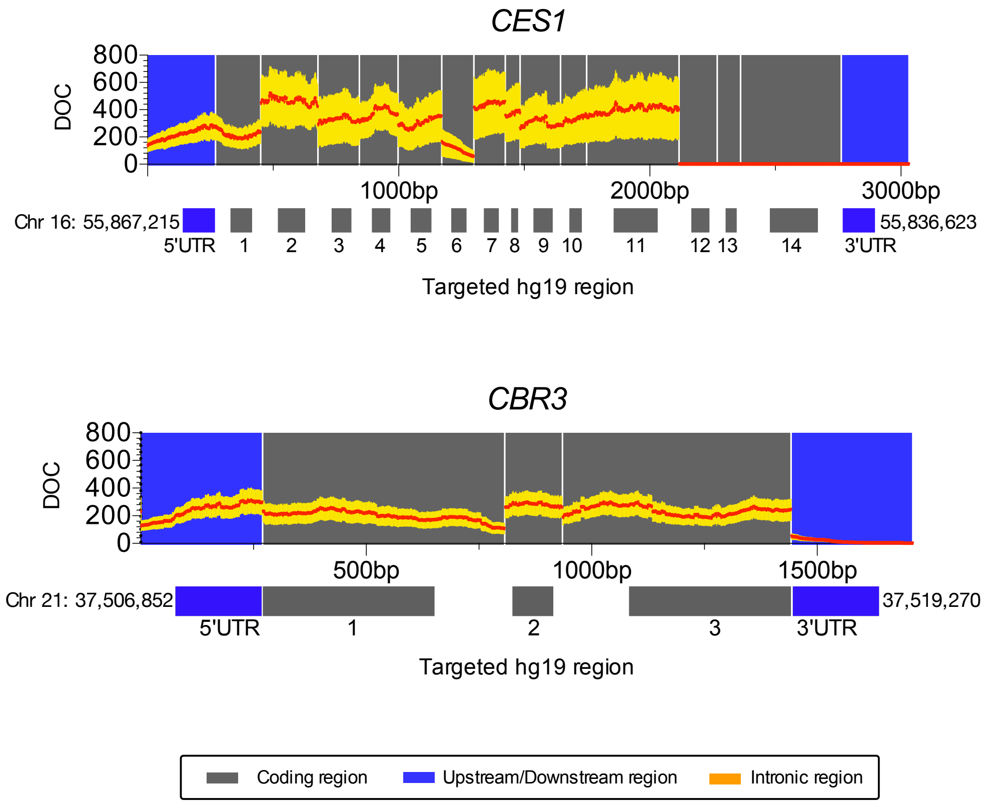

Figure S5. Study minor allele frequencies (MAF) in relation to the reported MAF in 1000 Genomes Project (1000G) and Exome Aggregation Consortium (ExAC) datasets.

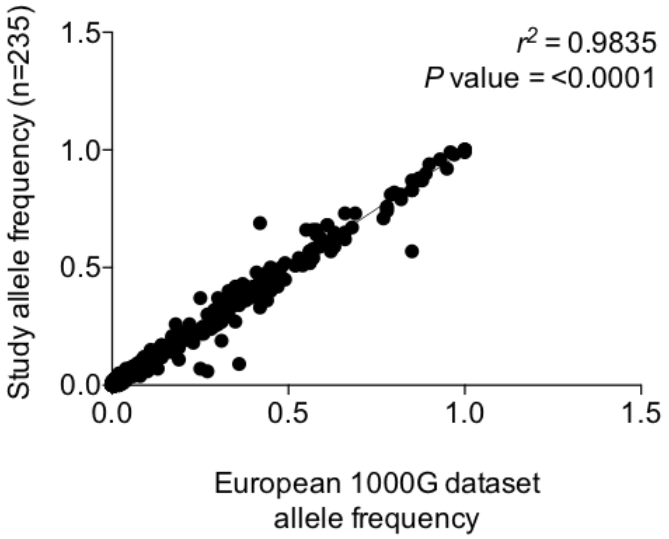

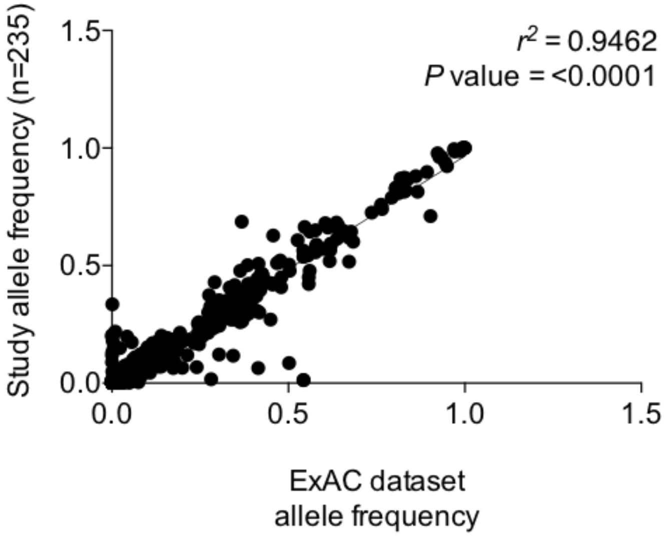

Figure S6. *In silico* functional prediction scores for genetic variants identified among 235 subjects. Rare or novel variations had a greater proportion of possibly deleterious prediction scores for all three algorithms (SIFT, Polyphen-2 and CADD).

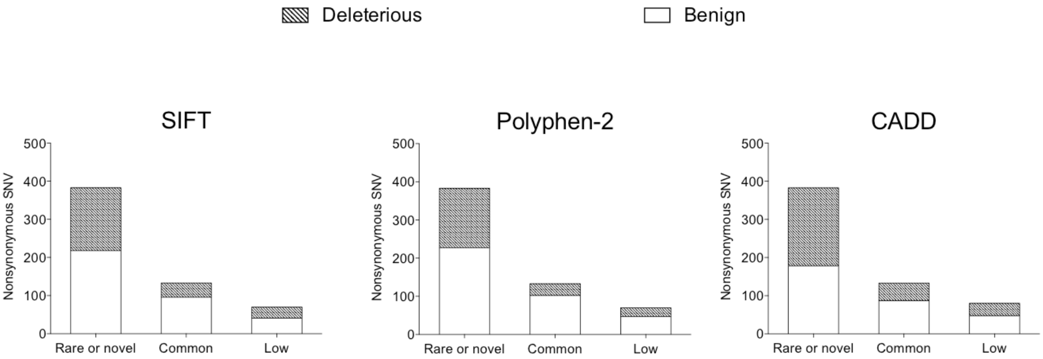

	SIFT Rare or novel	SIFT Common	SIFT Low	Polyphen-2 Rare or novel	Polyphen-2 Common	Polyphen-2 Low	CADD Rare or novel	CADD Common	CADD Low
Deleterious, n	184	38	29	157	26	24	200	44	31
Benign, n	199	100	41	225	112	46	190	96	42
Deleterious, %	48.0%	27.5%	41.4%	41.1%	18.8%	34.3%	51.3%	31.4%	42.5%
Chi-squared, *P* value		0.0002			< 0.0001			0.0002	

Figure S7. Zygosity of the potentially deleterious variants (CADD scaled score greater than 20) per subject (n = 235), showing there were more heterozygous compared to homozygous variants per subject .

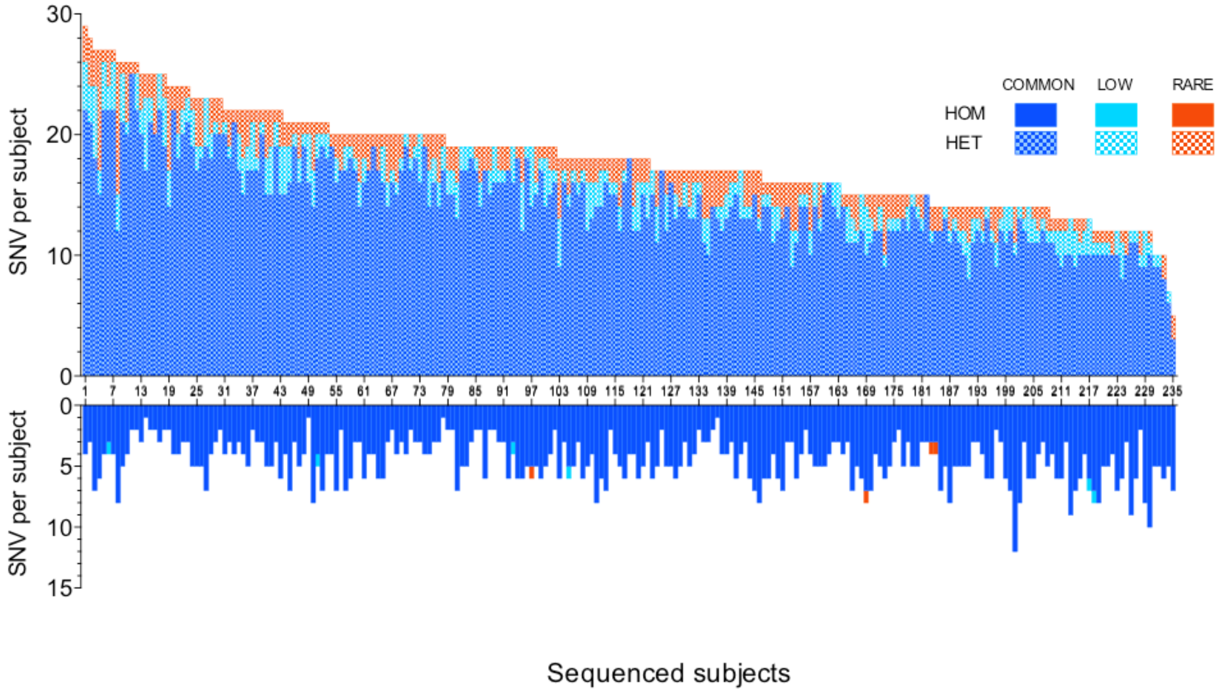

Figure S8. Single nucleotide variants (SNV) per subject (n = 235) found in cytochrome P450 (CYP) enzymes (*CYP1A1, CYP1A2, CYP1B1, CYP2B6, CYP2C19, CYP2C8, CYP2C9, CYP2D6, CYP2J2, CYP3A4, CYP3A5*, and *CYP4F2*) that are potentially deleterious variants (CADD scaled score greater than 20) separated by zygosity.

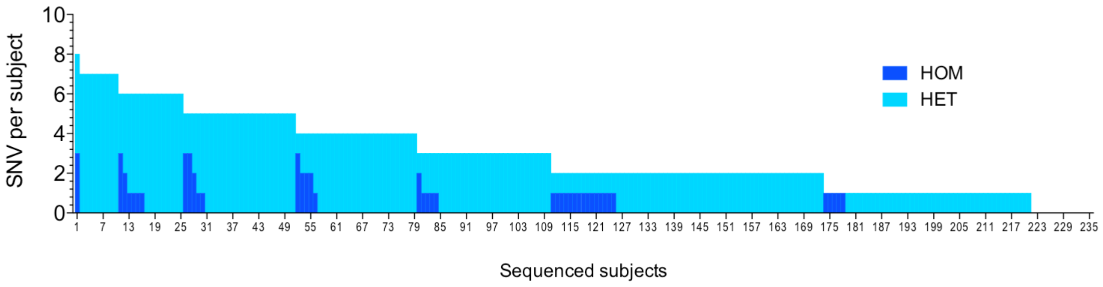

Figure S9. Number of Pharmacogenomics Knowledge Base (PharmGKB) “Level 1A/1B” variants (categorized as having strong supporting evidence for affecting drug efficacy/response and/or specific prescribing recommendations https://www.pharmgkb.org/clinicalAnnotations) found in 235 subjects separated by zygosity.

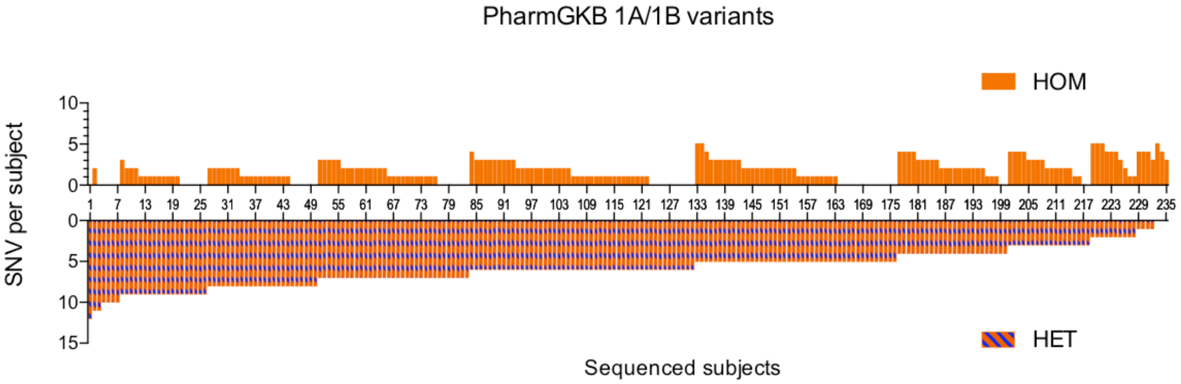

Figure S10. Histogram of the *GSTM1* and *GSTT1* gene coverage as a fraction total subject coverage in 235 subjects.

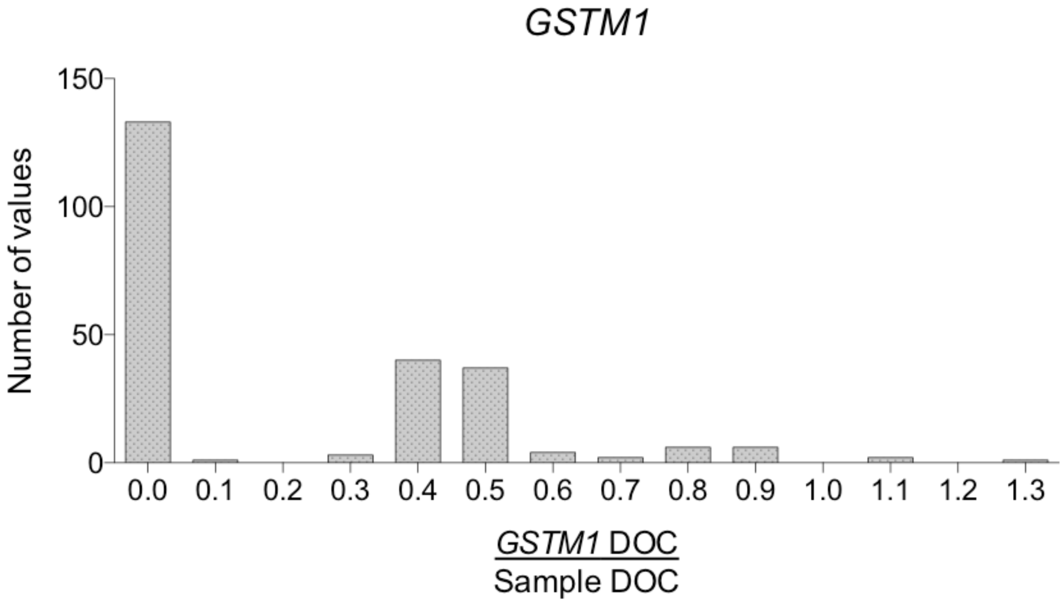

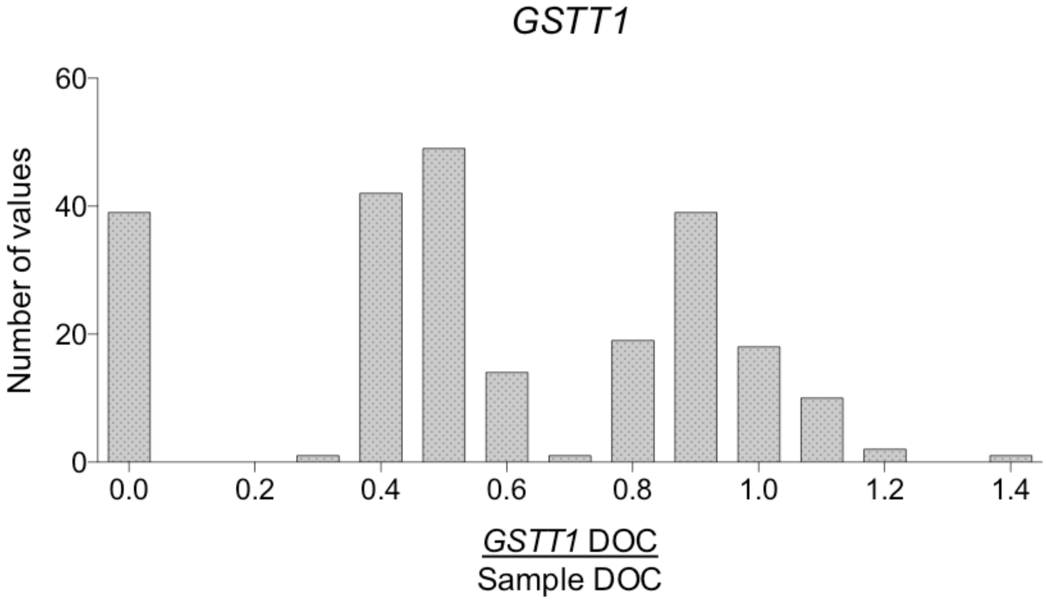

Supplement: Supplementary file 2 — Figure S1. Mean (±SD) read count per subject (duplicates removed) stratified by sequencing cluster (n = 246). Figure S2. Number of reads versus mean base quality score (Phred scale) per read for all sequencing runs (n = 246). Figure S3. Assessment of guanine and cytosine [GC] content within sequencing reads (n = 246). Histogram of the average percent GC content across total reads (A). Relationship between subjects average GC content and coverage (B). Figure S4. Mean (±SD) depth of coverage (DOC) across the targeted sequence for CES1 and CBR1 showing the inaccessible target regions. Figure S5. Study minor allele frequencies (MAF) in relation to the reported MAF in 1000 Genomes Project (1000G) and Exome Aggregation Consortium (ExAC) datasets. Figure S6. In silico functional prediction scores for genetic variants identified among 235 subjects. Rare or novel variations had a greater proportion of possibly deleterious prediction scores for all three algorithms (SIFT, Polyphen-2 and CADD). Figure S7. Zygosity of the potentially deleterious variants (CADD scaled score greater than 20) per subject (n = 235), showing there were more heterozygous compared to homozygous variants per subject. Figure S8. Single nucleotide variants (SNV) per subject (n = 235) found in cytochrome P450 (CYP) enzymes (CYP1A1, CYP1A2, CYP1B1, CYP2B6, CYP2C19, CYP2C8, CYP2C9, CYP2D6, CYP2J2, CYP3A4, CYP3A5, and CYP4F2) that are potentially deleterious variants (CADD scaled score greater than 20) separated by zygosity. Figure S9. Number of Pharmacogenomics Knowledge Base (PharmGKB) “Level 1A/1B” variants (categorized as having strong supporting evidence for affecting drug efficacy/response and/or specific prescribing recommendations https://www.pharmgkb.org/clinicalAnnotations) found in 235 subjects separated by zygosity. Figure S10. Histogram of the GSTM1 and GSTT1 gene coverage as a fraction total subject coverage in 235 subjects. (PDF 2439 kb) [file 12920_2019_527_MOESM2_ESM.pdf]
